# Dog allergen-induced asthma in mice: a relevant model of T2^low^ severe asthma with airway remodelling

**DOI:** 10.1007/s00011-025-02004-9

**Published:** 2025-03-14

**Authors:** Victor Margelidon-Cozzolino, Joanne Balsamelli, Julie Carrard, Saliha Ait Yahia, Marie-Hélène Gevaert, Silvia Demoulin-Alexikova, Muriel Pichavant, Anne Tsicopoulos, Cécile Chenivesse, Stéphanie Lejeune, Patricia de Nadai

**Affiliations:** 1 Univ. Lille, CNRS, Inserm, CHU Lille, Institut Pasteur de Lille, U1019 - UMR9017 - CIIL-Center for Infection and Immunity of Lille, 59000 Lille, France; 2 Groupement Des Hôpitaux de L’Institut Catholique de Lille (GHICL), Lille, France; 3 Univ. Lille, CNRS, Inserm, CHU Lille, Institut Pasteur de Lille, US 41-UAR 2014-PLBS, Lille, France; 4 CRISALIS (Clinical Research Initiative In Severe Asthma: a Lever for Innovation & Science), F-CRIN Network, INSERM US015, Toulouse, France; 5 Univ. Lille, Department of Pediatric Pulmonology and Allergy, Hôpital Jeanne de Flandre, CHU Lille, 59000 Lille, France; 6 Service de Pneumologie, Hôpital Saint-Philibert, Rue du Grand But, 59160 Lomme, France

**Keywords:** Asthma, Dog allergen, Airway remodelling, Th17 inflammation, Neutrophils, Steroids

## Abstract

**Objective and design:**

Airway remodelling (AR) is a disabling phenomenon in patients with severe asthma, yet suitable models are lacking. We previously developed a dog allergen-induced murine asthma model characterized by T2^low^ Th17-driven neutrophilic airway inflammation and AR. To assess its relevance to human AR associated with T2^low^ severe asthma, a condition characterised by poor response to inhaled steroids, we tested the steroid sensitivity of the key features of this model.

**Material:**

Asthma was induced in C57BL/6 J mice by intranasal sensitization, followed by a three-week challenge with dog allergen. *Treatment*: Daily intraperitoneal 1 mg kg^−1^ dexamethasone was administrated during the last week of challenge. *Methods*: We measured airway resistances in response to methacholine, cellular inflammation in bronchoalveolar lavage, lung cytokines, and quantified AR features, in response to dexamethasone.

**Results:**

Dexamethasone-treated mice showed persistent airway hyperresponsiveness, neutrophilic inflammation, and *Il17*a overexpression, whereas *Il22* expression was abrogated. Pathological AR features, including mucus hyperproduction, subepithelial fibrosis and smooth muscle hypertrophy were not eliminated by dexamethasone.

**Conclusions:**

Our dog allergen-induced murine model of asthma mirrors the steroid-insensitive traits of human severe T2^low^ asthma with AR, making it a relevant tool for identifying novel therapeutic targets in this orphan asthma subset.

**Supplementary Information:**

The online version contains supplementary material available at 10.1007/s00011-025-02004-9.

## Introduction

Asthma is the most frequent chronic inflammatory pulmonary disorder, affecting millions of people worldwide. It is characterised by recurrent paroxysmal respiratory symptoms and variable airflow limitation, induced by airway inflammation and hyperresponsiveness (AHR) [1]. Around one third of people with asthma also experience permanent manifestations due to persistent airflow limitation [2], leading to exercise dyspnoea, respiratory disability and impaired quality of life [3, 4]. Persistent airway obstruction is the result of airway remodelling (AR) [4], corresponding to long-term pathological changes in bronchial mucosa, including mucus hyperproduction, airway smooth muscle hypertrophy and subepithelial fibrosis, which lead to thickened airway walls [5]. In most patients, inhaled corticosteroids (ICS) reduce airway inflammation and some components of AR, thereby improving symptom control [6]. In up to 10% of cases, asthma is steroid-insensitive and classified as severe asthma. Severe asthma is defined by the need of high-dose ICS plus another controller to achieve satisfactory disease control, or by the use of oral steroids more than half a year, despite good adherence to treatment and management of modifiable contributory factors [7–9]. Around half of patients with severe asthma have persistent airflow obstruction [10]. Additionally, persistent airflow obstruction is associated with asthma severity and steroid unresponsiveness [2, 11].

The need for alternative drugs to steroids in severe asthma has led to the development of biologics, monoclonal antibodies targeting specific inflammatory molecules [12]. However, these therapies are mainly effective in T2^high^ severe asthma, i.e., involving eosinophilic inflammation and Th2 cytokines (mainly Interleukin (IL)-4, IL-5 and IL-13). They effectively reduce the risk of severe exacerbations and the need for oral steroid use, while their impact on airflow limitation and AR is minimal [13]. Patients with severe T2^low^ asthma, frequently associated with neutrophilic and Th17 inflammation (mainly involving IL-17 and IL-22), have currently limited therapeutic options [14]. Only the anti-TSLP biologic, tezepelumab, has been approved in this setting with, however, a poorer response than in patients with T2^high^ asthma [15]. Additionally, T2^low^ asthma patients seem more prone to develop AR as they exhibit poorer lung function than T2^high^ asthma patients [16] and as some hallmarks of T2^low^ inflammation, such as sputum neutrophilia, IL-17 and IL-22 levels, are particularly correlated to AR [17, 18].

The subset of patients with AR has currently no effective treatment option apart from bronchial thermoplasty, an interventional procedure approved for patients with substantial AR [19]. The lack of effective therapy for AR is related to our still-limited understanding of its pathobiological processes. Only scarce asthma animal models succeeded in generating AR, mostly in T2^high^ settings. To address this issue, we have developed an original murine model of asthma, based on dog allergen (DOG) intranasal administration, reproducing pathological features of AR, along with neutrophilic and predominantly Th17-driven airway inflammation [20]. Nevertheless, the steroid responsiveness of asthma outcomes, which defines asthma severity in humans, and particularly that of AR features in this model, remains unknown.

To evaluate whether our DOG-induced murine model of asthma accurately mimics human T2^low^ severe asthma with AR, and thus is suitable to investigate underlying mechanisms and potential therapeutic targets, we assessed the response to steroids of the main features of this model, including AHR, neutrophilic and Th17 inflammation, and AR.

## Material and methods

### Mice

All experiments were performed in C57BL/6 J female mice (6 weeks of age), purchased from Janvier Lab (Le Genest-Saint-Isle, France), housed under standard pathogen-free conditions, with ad libitum access to food and water. Animal experiments were conducted in an accredited establishment at the Institut Pasteur de Lille, France, in accordance to governmental guidelines and the European directive 2010/63/EU, approved by the local ethical committee and authorized by the ministry of research and innovation (N° APAFIS#38135-2022072615498543 v7).

### Material and reagents

Dog allergen extract was kindly provided by ALK France, as dry powder, reconstituted and diluted in filtered phosphate-buffered saline (PBS, Fisher Scientific, Courtaboeuf, France) buffer. Once reconstituted and diluted, the final concentration of lipopolysaccharide in the solution administered to mice was measured to be 0.08 ng.mL^−1^. Dexamethasone was purchased from Sigma-Aldrich (St Louis, MO, USA, reference: D4902), as dry powder. Dexamethasone was first reconstituted at 10 µg.µL^−1^ in ultrapure ethanol, and then diluted at 0.15 µg.µL^−1^ in PBS for intraperitoneal administration.

### Experimental protocol

**Fig. 1:**
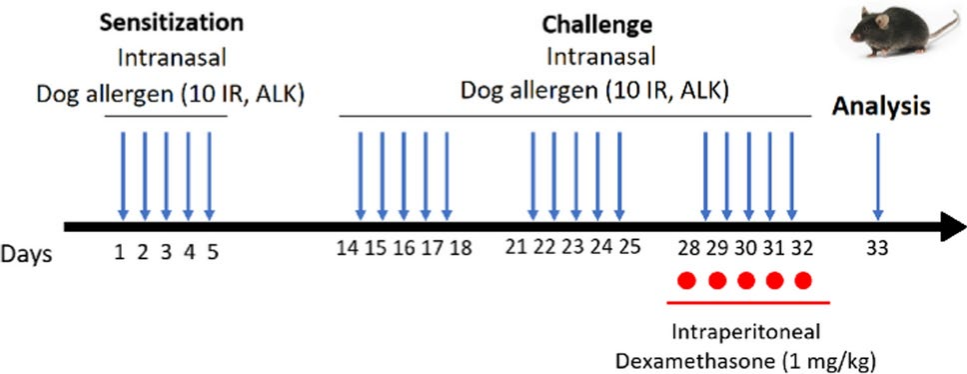
Experimental protocol assessing the response to DEX in DOG-induced murine model of asthma. DOG (10 IR) or PBS was intranasally administered in 6-week-old C57BL/6 J mice for 5 days in the first phase of sensitization. After a nine-day break, mice were intranasally challenged with DOG again 5 days a week during 3 consecutive weeks. During the last week of challenge, 1 mg.kg^−1^ DEX or PBS was intraperitoneally administered to mice 30 min before the intranasal challenge

After isoflurane anaesthesia, mice were sensitized by intranasal administration of 30 µL of DOG solution (equivalent to a dose of 10 IR (Index of Reactivity), solution titrated at 10 µg.mL^−1^ of major allergen Can f 1) or Phosphate Buffer Saline (PBS) for 5 consecutive days. Nine days later, mice were challenged with the same quantity of DOG or PBS, for 3 consecutive weeks (5 days a week) (Fig. 1). During the last challenge week, mice randomly received intraperitoneal dexamethasone (DEX, 1 mg.kg^−1^, achieved by administering 6.67 µL.g^−1^ body weight of a DEX solution titrated at 0.15 µg.µl^−1^) or PBS (6.67 µL.g^−1^ body weight) 30 min before intranasal challenge. Twenty-four hours after the last challenge, mice were euthanized with intraperitoneal Euthasol (140 mg.kg^−1^ body weight). Bronchoalveolar lavage (BAL) fluids, blood and lung tissue were collected for analyses. For AHR assessment, some mice were intraperitoneally anesthetized with 5 mL.kg^−1^ of a 10% medetomidine/10% ketamine solution. Mice were categorized into 4 groups according to intranasal/intraperitoneal exposures: PBS/PBS, PBS/DEX, DOG/PBS and DOG/DEX.

### AHR assessment

Airway resistances were measured in response to increasing doses of nebulized methacholine (0–100 mg.mL^−1^ in PBS) and analysed using Flexivent® (Scireq®, Montreal, Canada) as previously described [21]. Results are expressed as the difference between the maximal resistance value measured at the corresponding dose of methacholine and the baseline maximal resistance value with no methacholine (${\Delta Rrs}_{max}$).

### BAL supernatant and cell collection

BAL was performed in mice assessed for AHR, immediately after exsanguination, using 500 µL of ice-cold PBS for supernatant analysis and 1 ml more for cytological analysis, injected and aspirated through the intratracheal catheter used for Flexivent®. After soft centrifugation (300 g, 7 min, 4 °C), the supernatant of the 500 µL lavage was collected and cells from the total 1500 µL lavage were suspended in 100 µL of PBS for total leukocyte number count. The number of cells in 0.1 µL samples was determined using a Thoma counting chamber under × 40 magnification. After cytocentrifugation of 100,000 cells, and May-Grünwald Giemsa (MGG) staining, macrophages, eosinophils, neutrophils and lymphocytes were identified and enumerated under × 20 magnification. Leukocyte subtypes were enumerated on cytospots containing at least 300–400 cells whenever possible. The differential cell count for each leukocyte subtype was calculated as the proportion of each subtype relative to the total number of cells counted. The total number of each leukocyte subtype was then obtained by multiplying the percentage for each subtype by the total number of cells determined prior to cytocentrifugation.

### Lung histology and immunohistochemistry

Unwashed left lungs were fixed in Antigenfix and embedded in paraffin according to the manufacturer’s indications. Embedded lungs were then cut (5 µm) with a Microtom (Microm HM355S, Thermoscientific) and stained with Haematoxylin and Eosin (H&E) for airway inflammation, Periodic Acid-Schiff (PAS) for airway mucus production assessment, and with Masson’s trichrome for subepithelial fibrosis assessment. Mucus production and subepithelial fibrosis were quantified using semi-quantitative scores for each bronchus (Supp. Tab. 1 & 2), assessed under × 20 magnification, according to a previously published method [20]. Data were expressed as the mean score per bronchus.

To quantify smooth muscle cells area, an immunohistochemical method of staining, using a monoclonal mouse anti-α-smooth muscle actin (α-SMA) antibody (clone 1A4) and a mouse-on-mouse kit-AP detection system with permanent Red, was used. The red stained area of SMC was measured using ImageJ software (Rasband, W.S., ImageJ, U.S. National Institutes of Health, Bethesda, Maryland, USA) on micrographs of each bronchus taken at × 20 magnification. Results were expressed as µm^2^ surface area per µm of bronchial basement.

Histological scores and smooth muscle quantification were were analyzed in a blinded manner with respect to group allocation. For all histological analyses, every bronchus from three lung sections representative of the upper part, the middle part, and the lower part of the left pulmonary lobe, were assessed.

### Protein analysis

Quantifications of dog-specific Immunoglobulin (Ig) E in serum and IgG_1_ in BAL were performed by ELISA as previously described [20], by coating 96-well plates with 10 µg.mL^−1^ of DOG solution diluted in PBS. Dog-specific IgE and IgG_1_ antibodies were detected by biotinylated goat anti-IgE or -IgG_1_ respectively. Binding of Streptavidin-Horse Radish Peroxidase was revealed by TMB substrate solution and the OD value at 450 nm was determined. Total IgA and MPO levels in BAL fluid were quantified using commercial ELISA kits following the manufacturer's instructions. IL-4, IL-13, IL-17A, and CXCL1 levels were quantified in total lung tissue, after protein extraction, using a multiplex ELISA kit, according to the manufacturer's instructions. Data were normalized to total lung protein content.

### RNA isolation and quantitative RT-PCR

RNA extraction was processed from lungs with a Nucleospin RNA mini kit (Macherey–Nagel, Hoerdt, France) according to the manufacturer’s instructions. Extracted RNA was reverse-transcribed with the High-Capacity cDNA Archive kit (Applied Biosystems, Foster City, USA), according to the manufacturer’s instructions. cDNA was then amplified using the IDT Prime Time Assay master mix. Real Time-PCR was performed with Prime Time Assay primers (IDT, Leuven, Belgium) and detected on a QuantStudio 12 K flex (Applied Biosystems, Waltham, USA). Data were analysed with the ThermoFisher cloud. Relative mRNA levels (2^−∆∆Ct^) were determined by comparing the PCR cycle thresholds (Ct) for the gene of interest and *Rplp0* (∆Ct), and ∆Ct values for treated and control groups (∆∆Ct). The primers (Integrated DNA technologies) used for RT-qPCR are shown in Supp. Tab. 3. *Rplp0* (Ribosomal Protein Lateral Stalk Subunit P0) was used as internal reference gene to normalize the transcript levels.

### Statistical analysis

All experiments were carried out at least twice. Normally distributed data (Shapiro–Wilk normality test) were analysed by one-way analysis of variance (ANOVA), followed by post-hoc multiple comparison test (Dunn’s test). Non-normally distributed data were analyzed using Kruskal–Wallis nonparametric tests with Dunn’s post-hoc tests in GraphPad Prism (GraphPad software v9, Inc., La Jolla, CA, USA). A *p*-value < 0.05 was considered statistically significant. For AHR, a dose response curve was performed and groups were compared using a two-way ANOVA test. For all analyses, outliers were excluded using Grubbs' test. Only statistically significant results (*p* < 0.05) are shown in the figures for clarity. Non-significant results are omitted, but relevant findings are discussed in the text. Data represent a pool of mice from 4 independent experiments.

Unmentioned details for purchased reagents, including suppliers, and for histological scorings are provided in Supplementary data S1.

## Results

### DEX does not reduce DOG-induced AHR

**Fig. 2:**
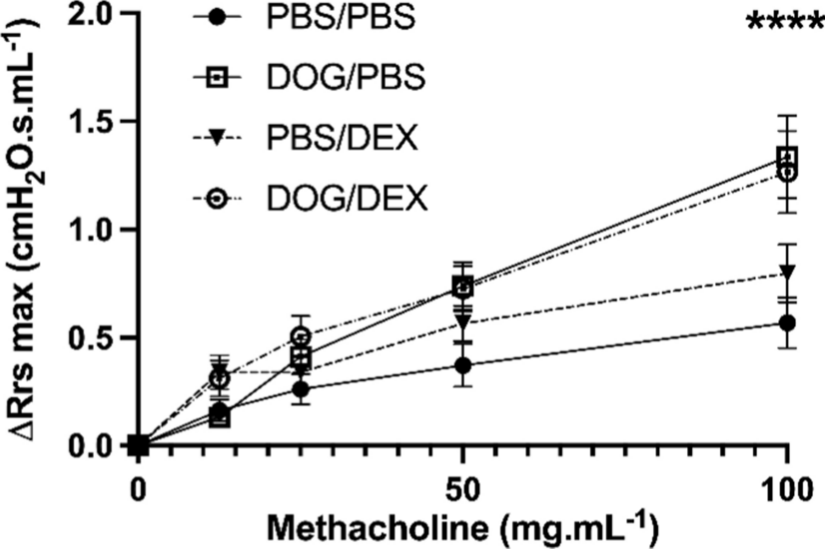
Assessment of AHR. Increase in airway resistances from basal value (${\Delta Rrs}_{max}$) in response to 0–100 mg.mL^−1^ doses of nebulized methacholine, measured with Flexivent® by tracheal canulation. Data are expressed as mean ± SEM; n = 5–19 per group; Two-way analysis of variance test, ****p < 0.0001 for DOG/PBS vs PBS/PBS and DOG/DEX vs PBS/PBS at 100 mg.mL^−1^, *p* > 0.05 for all other comparisons. Experiments were performed 3 times

AHR is an important contributor to asthma pathophysiology. We already reported the induction of AHR by the exposure to DOG in our model [20]. In order to determine whether this feature was affected by steroids, airway resistances in response to increasing doses of methacholine were measured 24 h after the last intranasal challenge. Mice from the DOG/DEX group showed a similar increase in airway resistances as those from the DOG/PBS group (*p* > 0.05) (Fig. 2). These data show that AHR is insensitive to DEX in our model.

### Despite reduction, DEX does not abrogate DOG-induced neutrophilic airway inflammation

**Fig. 3:**
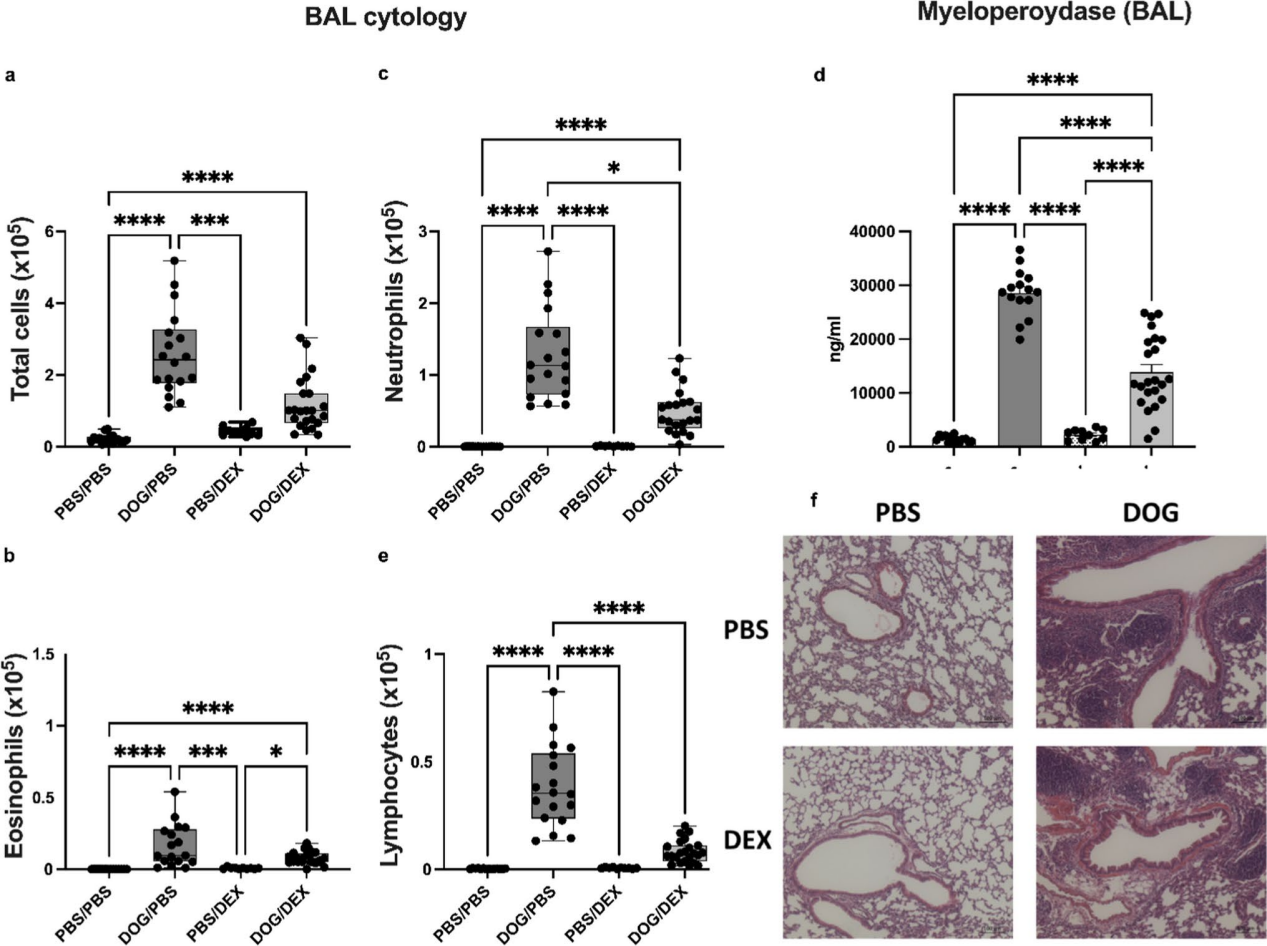
Assessment of airway inflammation in BAL. **a**-**c** & **e**: Cytological analysis of total leukocytes and leukocyte subtypes in BAL. **a**: total leukocyte number. **b**: total eosinophil number; **c**: total neutrophil number; **e**: total lymphocyte number. Data are expressed as median, interquartile range, minimum and maximum; n = 9–23 per group; Kruskall-Wallis tests, *p < 0.05, ***p < 0.001, ****p < 0.0001. **d**: quantification of MPO in BAL by ELISA. Data are expressed as mean ± SEM; n = 10–23 per group; Ordinary one-way analysis of variance test, ****p < 0.0001. Ruled-out outliers: for a: no outlier; for b: 2 outliers in PBS/PBS, 1 in DOG/PBS, 2 in DOG/DEX; for c: 2 outliers in PBS/PBS, 1 in PBS/DEX, 2 in DOG/DEX; for d: 2 outliers in PBS/PBS; for e: 2 outliers in PBS/PBS. f: Representative photographs of H&E-stained lung sections, scale bars 100 µm. Experiments were performed 3 times

As previously reported, this DOG-induced model of asthma is characterized by prominent neutrophilic airway inflammation [20]. To investigate the steroid sensitivity of this parameter, the effect of DEX on BAL cells was assessed. The total leukocyte count was significantly higher (*p* < 0.0001) in the DOG/PBS group compared to the PBS/PBS group (Fig. 3a). BAL cell number was not significantly different in DOG/DEX mice compared to the DOG/PBS group (*p* = 0.0511, Fig. 3a). Total cell counts still remained higher in DOG/DEX mice than in PBS/DEX (*p* = 0.16) and PBS/PBS mice. After MGG staining, similar results were obtained for eosinophil number in BAL (Fig. 3b).

As previously described, neutrophils were the main cell type increased in BAL in DOG/PBS mice compared to PBS/PBS mice (*p* < 0.0001) [20]. Interestingly, their number was significantly decreased by DEX (*p* = 0.04) but was not abolished, with a higher level in the DOG/DEX group compared to the PBS/DEX (*p* = 0.07) and PBS/PBS groups (Fig. 3c). To confirm this result, mouse MPO, mainly expressed in neutrophils, was measured in BAL fluids and showed consistent results (Fig. 3d). Regarding lymphocytes and macrophages counts, DEX treatment was associated with a dramatic reduction in DOG/DEX mice reaching similar levels to PBS/DEX (respectively *p* = 0.23 for lymphocytes and *p* = 0.86 for macrophages) and PBS/PBS groups (Fig. 3e, data not shown for macrophages). Taken together, these results show that DOG-induced airway neutrophilia is partially inhibited but persists despite steroid administration, demonstrating incomplete response to steroid of neutrophilic inflammation.

### DEX does not reduce DOG-induced Il17a overexpression but abrogates Il22 expression in lungs

**Fig. 4:**
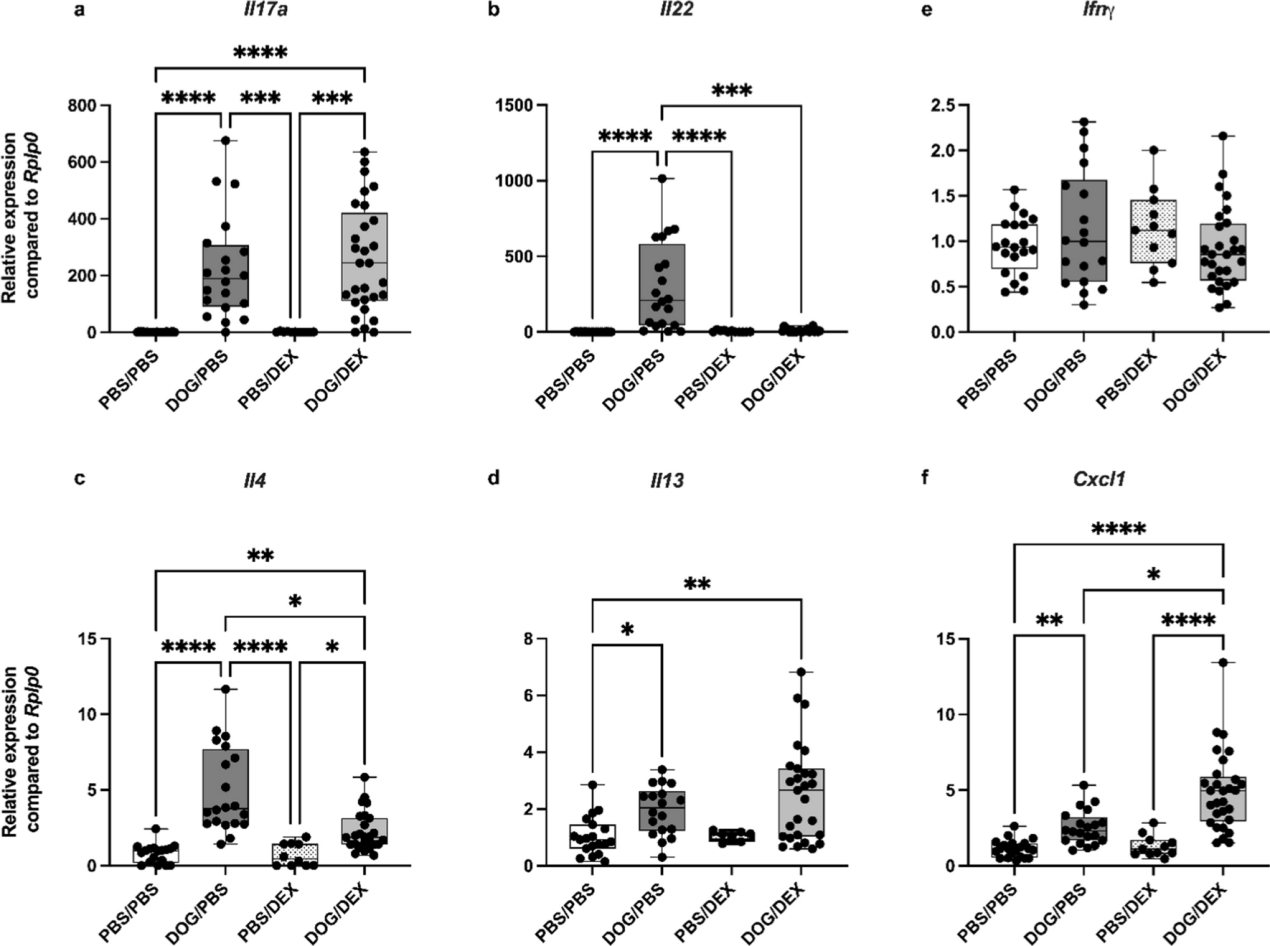
Assessment of cytokine and chemokine gene expressions in total lung tissue. **a**: *Il17a*. **b**: *Il22*. **c**: *Il4*. **d**: *Il13*. **e**: *Ifnγ.***f**: *Cxcl1*. Results are expressed as relative expression compared to housekeeping *Rplp0* gene expression, median, interquartile range, minimum and maximum; n = 7–29 per group; Kruskall-Wallis tests, *p < 0.05, **p < 0.01; ***p < 0.001, ****p < 0.0001. Ruled-out outliers: for a: 1 outlier in PBS/PBS, 1 in DOG/PBS; for b: 3 outliers in PBS/PBS, 1 in DOG/PBS, 5 in DOG/DEX; for c 1 outlier in PBS/PBS, 1 in DOG/PBS, 2 in DOG/DEX; for d: 2 outliers in PBS/PBS, 3 in DOG/PBS, 2 in PBS/DEX, 2 in DOG/DEX, for e: 1 outlier in PBS/PBS, 3 in DOG/PBS, 1 in DOG/DEX; for e: 1 outlier in DOG/PBS. Experiments were performed 4 times

In the DOG-induced model of asthma, airway inflammation is mainly driven by Th17 cytokines (IL-17A and IL-22) [20]. To assess the effect of DEX on this feature, the expression levels of polarizing cytokines and the neutrophil-chemoattractant CXCL1 were quantified by RT-qPCR in total lung tissue. Interestingly, the increase in *Il17a* expression in the DOG/PBS group compared to PBS/PBS group (*p* < 0.0001) was not modified by DEX (*p* > 0.99) in DOG/DEX mice (Fig. 4a). Similar results were observed for *Il17f* (data not shown). Conversely, the overexpression of* Il22* observed in the DOG/PBS mice (*p* < 0.0001 in comparison to PBS/PBS mice) was completely abolished by DEX (*p* = 0.0006), reaching comparable levels to those of PBS/DEX (*p* > 0.99) and PBS/PBS groups (Fig. 4b). In our model, expressions of Th2 cytokines *Il4* and *Il13* were less induced by DOG than those of Th17 cytokines, as previously reported. In DOG/DEX mice, *Il13* expression was similar to that of DOG/PBS group (*p* > 0.99) whereas *Il4* expression was significantly decreased (*p* = 0.02), although remaining significantly higher than in PBS/DEX (*p* = 0.03) and PBS/PBS mice (Fig. 4c and d). The Th1 cytokine *Ifn*$\gamma$ gene expression was not modulated by DOG (*p* = 0.63) and not modified by DEX (*p* = 0.43) (Fig. 4e). The neutrophil-attracting chemokine *Cxcl1* expression was significantly enhanced by DOG in DOG/PBS mice compared to PBS/PBS group (*p* = 0.0042) Fig. 4f). This increase was even significantly enhanced by DEX in DOG/DEX mice (*p* = 0.0393).

**Fig. 5:**
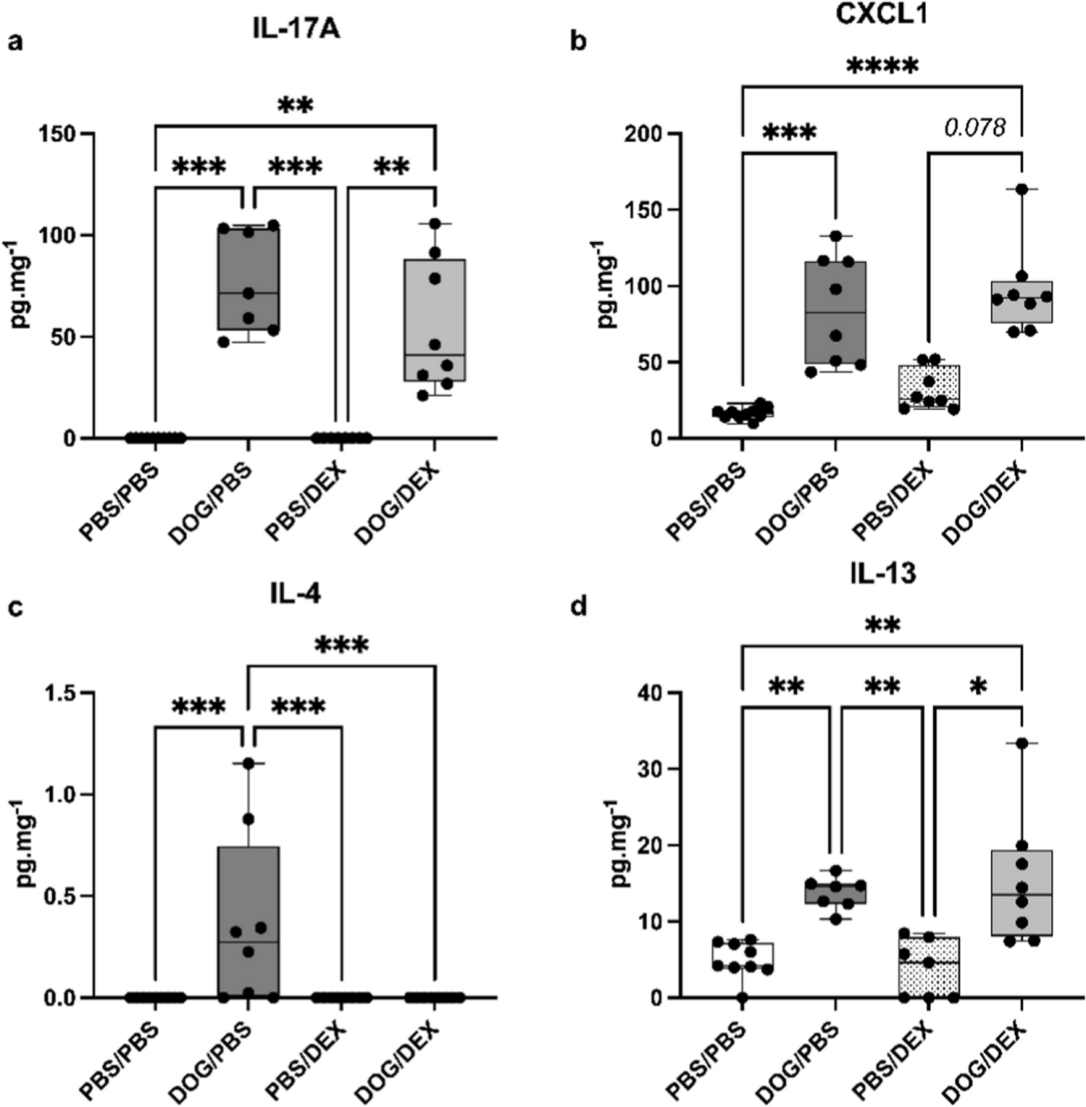
Assessment of cytokine and chemokine productions in lung tissue. **a**: IL-17A. **b**: CXCL1. **c**: IL-4. **d**: IL-13. Results are expressed in pg.mg^−1^ of total lung tissue proteins, as median, interquartile range, minimum and maximum; n = 7–10 per group; Kruskall-Wallis tests, *p < 0.05, **p < 0.01; ***p < 0.001, ****p < 0.0001. Ruled-out outliers: for a: 1 outlier in DOG/PBS; for b: no outlier; for c 1 outlier in PBS/PBS, for d: no outlier. Experiments were performed 4 times

To further validate these gene expression findings, we additionally quantified the production of IL-17A (Fig. 5a), CXCL1 (Fig. 5b), IL-4 (Fig. 5c), and IL-13 (Fig. 5d) in total lung tissue homogenates. The results were consistent with those obtained by PCR. IL-17A and IL-13 productions were unaffected by DEX (*p* > 0.99 for both, comparing DOG/DEX mice to DOG/PBS mice) while IL-4 production was abrogated by DEX (*p* = 0.0009 for DOG/DEX mice in comparison to DOG/PBS mice, *p* > 0.99 in comparison to PBS/DEX mice). CXCL1 levels remained unchanged (*p* > 0.99 for DOG/DEX mice in comparison to DOG/PBS mice). Overall, these data suggest that, pro-inflammatory Th17 and Th2 inflammatory responses remain active despite the administration of DEX, except for *Il22* expression which is fully responsive to DEX.

### DEX does not abrogate DOG-induced immune humoral response

**Fig. 6:**
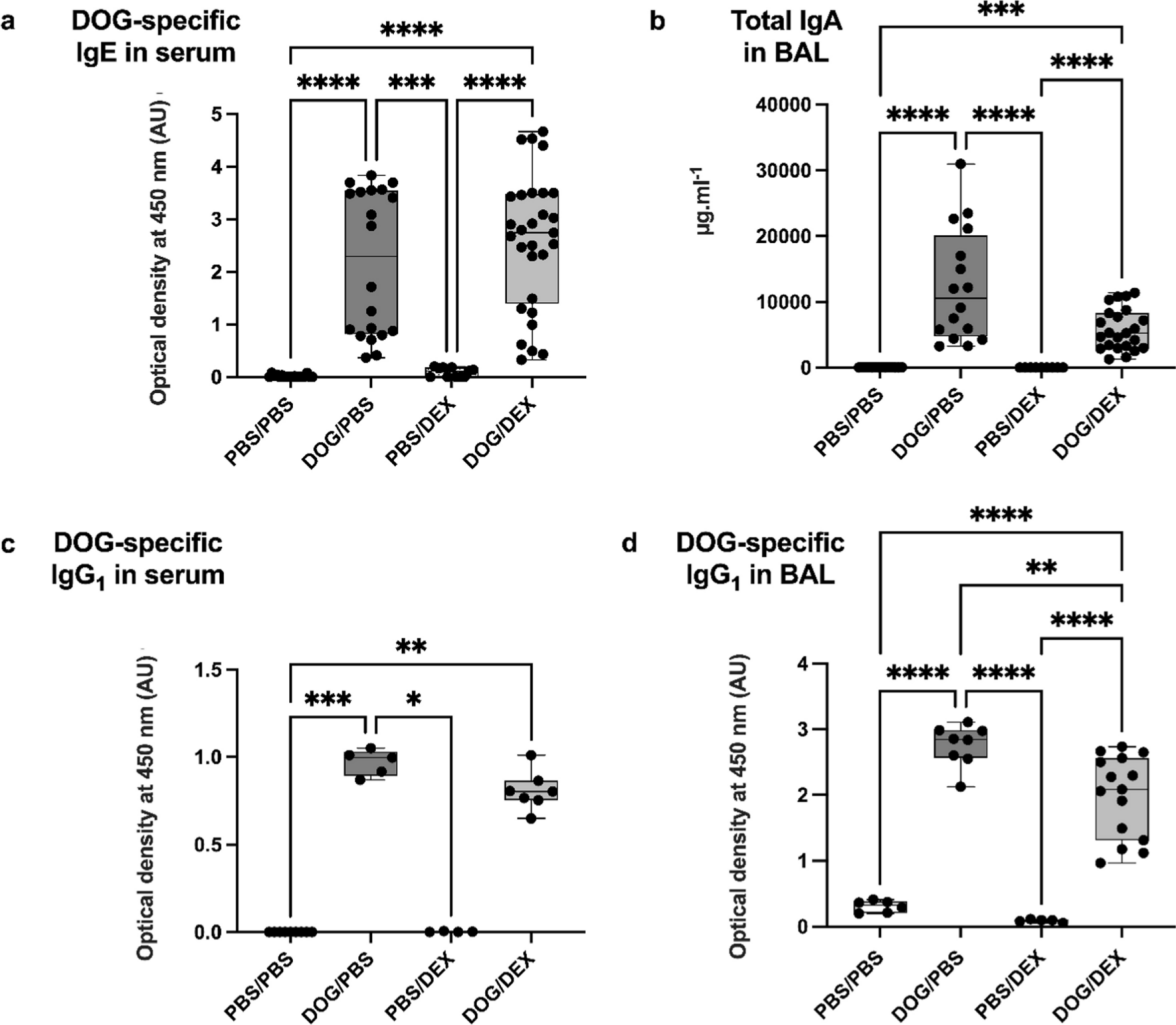
Assessment of systemic and lung humoral responses. Ig were measured by ELISA in serum and BAL. **a**: Quantification of DOG-specific IgE in serum (n = 11–29 per group). **b**: Quantification of total IgA in BAL (n = 9–23 per group). **c**: Quantification of DOG-specific IgG_1_ in serum (n = 4–9 per group). **d**: Quantification of DOG-specific IgG_1_ in BAL (n = 6–15 per group). AU: arbitrary unit. Results are expressed as median, interquartile range, minimum and maximum; Kruskall-Wallis tests, *p < 0.05; **p < 0.01; ***p < 0.001, ****p < 0.0001. Ruled-out outliers: for a: 3 outliers in PBS/PBS; for b: 2 outliers in PBS/PBS, 1 in PBS/DEX; for c: 1 outlier in PBS/DEX; for d: no outlier. Experiments were performed 4 times for a & b, once for c, twice for d

As previously reported, the DOG-induced murine model of asthma is characterized by a marked systemic and local humoral response with enhanced production of DOG-specific IgE in serum, IgG1 and total IgA in BAL [20]. No significant modulation of serum DOG-specific IgE was observed in the DOG/DEX compared with the DOG/PBS group (*p* > 0.99, Fig. 6a). Total IgA in BAL were not statistically different in DOG/DEX mice compared to DOG/PBS mice (*p* = 0.58) and remained significantly higher compared with mice from PBS/DEX (*p* < 0.0001) and PBS/PBS groups (Fig. 6b). Additionally, DOG also induced a DOG-specific IgG_1_ response in serum (DOG/PBS vs PBS/PBS, *p* < 0.001), that was not reduced by dexamethasone (DOG/DEX vs DOG/PBS, *p* > 0.99). In contrast, DOG-specific IgG1 levels in BAL were significantly diminished by DEX (*p* = 0.0028) but remained markedly higher in the DOG/DEX mice than in the PBS/DEX (*p* < 0.0001) and PBS/PBS groups (Fig. 6c). Overall, these results demonstrate a persistent strong local and systemic humoral response despite DEX in the DOG-induced model.

### DEX does not abrogate AR in DOG-induced murine model of asthma

**Fig. 7:**
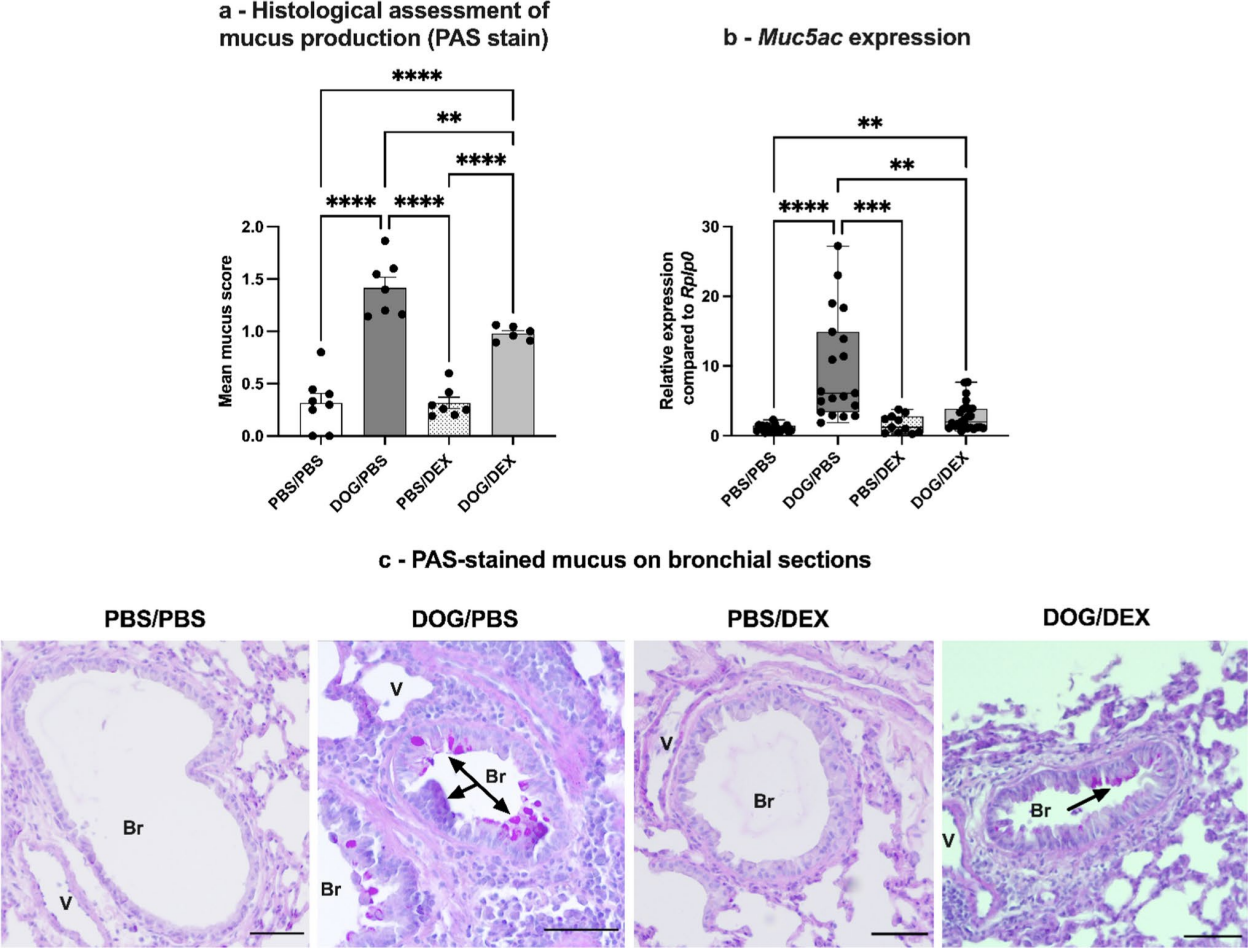
Assessment of mucus production**. a**: Semi-quantitative histological assessment of mucus production. Lung sections were stained with PAS. The intensity of mucus production was scored for each bronchus. Data are expressed as mean ± SEM, n = 6–8 per group; Ordinary one-way analysis of variance test, **p < 0.01, ****p < 0.0001. Ruled-out outliers: 1 outlier in DOG/PBS, 2 in DOG/DEX. **b**: Assessment of mucin gene *Muc5ac* expression by RT-qPCR in total lung tissue. Results are expressed as relative expression compared to housekeeping *Rplp0* gene expression, median, interquartile range, minimum and maximum; n = 11–29 per group; Kruskall-Wallis test, **p < 0.01; ***p < 0.001, ****p < 0.0001. Ruled-out outliers: 1 outlier in PBS/PBS, 2 in DOG/PBS, 4 in DOG/DEX. **c**: Representative photographs of PAS-stained (purple colour) lung sections, black arrows show stained mucus, V: vessel, Br: bronchus, scale bars 50 µm. Experiments were performed 3 times for a, 4 times for b

The DOG-induced murine model of asthma is characterized by the presence of AR including mucus hyperproduction, subepithelial fibrosis, and airway smooth muscle hypertrophy [20]. To investigate the effect of DEX on AR, standard histological stains were performed on lung sections: PAS staining mucins to visualize mucus production, and Masson’s trichrome marking collagen fibers to assess subepithelial fibrosis. For both stains, a semi-quantitative scoring was used, applying an intensity score to each bronchus per lung section. Results for mucus production are shown in Fig. 7. Histological assessment showed that mucus production, although significantly diminished by DEX in the DOG/DEX mice as compared with the DOG/PBS group (*p* = 0.0049), remained significantly higher than in the PBS/DEX (*p* < 0.0001) and PBS/PBS groups (Fig. 7a). The expression quantification of *Muc5ac,* the main mucin modulated in the DOG-induced model, showed consistent results, with a reduction (*p* = 0.0066) more marked than the one of the histological score (Fig. 7b).

**Fig. 8:**
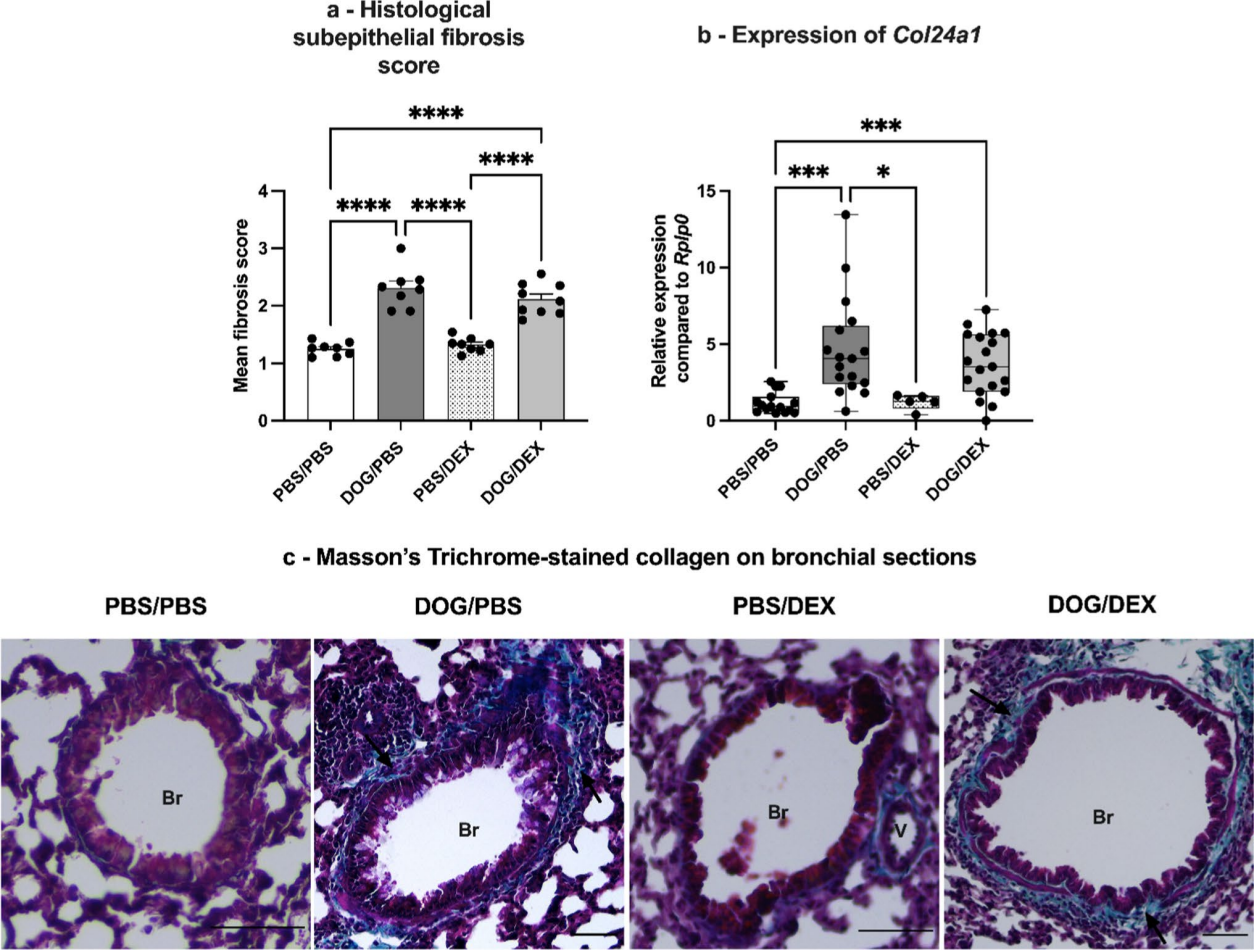
Assessment of subepithelial fibrosis. **a**: Semi-quantitative histological assessment of subepithelial fibrosis. Lung sections were stained with Masson’s trichrome. The severity of subepithelial fibrosis was scored for each bronchus. Data are expressed as mean ± SEM, n = 8–9 per group; Ordinary one-way analysis of variance test, ****p < 0.0001, no outlier ruled-out. **b**: Assessment of *Col24a1* expression by RT-qPCR in total lung tissue. Results are expressed as relative expression compared to housekeeping *Rplp0* gene expression, median, interquartile range, minimum and maximum; n = 5–19 per group; Kruskall-Wallis test, *p < 0.05; ***p < 0.001. Ruled-out outliers: 1 outlier in DOG/PBS. **c**: Representative photographs of Masson’s trichrome-stained (green color) lung sections, black arrows show stained collagen, V: vessel, Br: bronchus, scale bars 50 µm. Experiments were performed 3 times for a, 4 times for b

As shown in Fig. 8, the histological assessment of subepithelial fibrosis with Masson’s trichrome showed no modulation by DEX (*p* = 0.35, Fig. 8a). This result was supported by the expression quantification of *Col24a1,* coding for Collagen XXIV, a collagen type involved in tissue repair [22], in total lung tissue, which showed no reduction of DOG-induced overexpression in mice treated with DEX (*p* > 0.99, Fig. 8b).

**Fig. 9:**
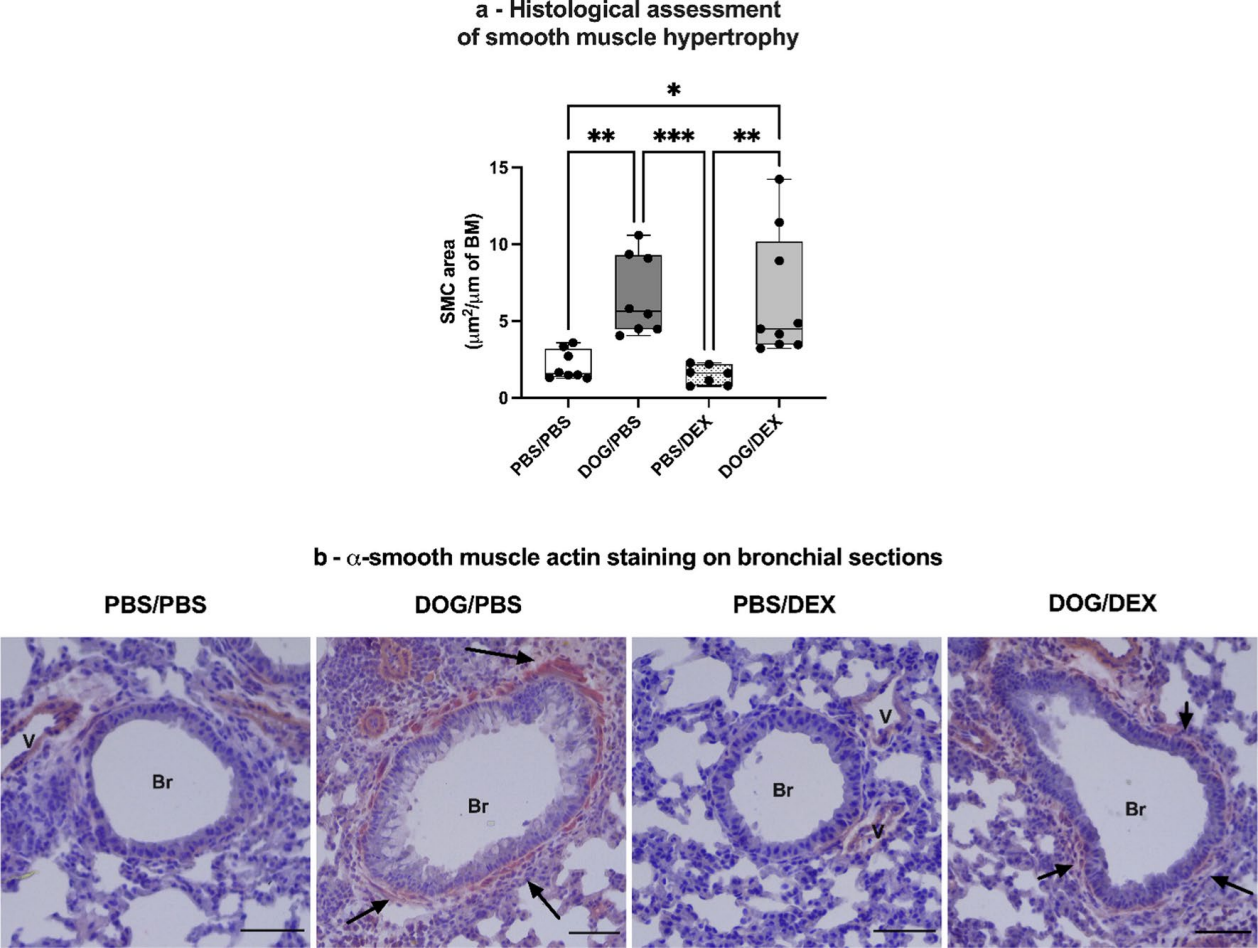
Assessment of smooth muscle hypertrophy. **a**. Lung sections were stained with anti $\alpha$-SMA antibody, staining smooth muscle cells. Red-stained area around each bronchus was measured and normalized with basement membrane circumference. Data are expressed as median, interquartile range, minimum and maximum; n = 7–9 per group; Ordinary one-way analysis of variance test, *p < 0,05; **p < 0,01; ***p < 0,001, ****p < 0,0001, no outlier ruled-out. **b**: Representative photographs of Immunostained smooth muscle cells (red color) on lung sections, black arrows show stained smooth muscle, V: vessel, Br: bronchus, scale bars 50 µm. Experiments were performed 3 times

To assess smooth muscle hypertrophy, an immunohistochemical staining of $\alpha$-smooth muscle actin, a specific structural protein of SMC, was applied to lung sections. A computed quantification of red-stained area around each bronchus was then performed. The increased stained area of SMC associated with DOG exposure was not reduced by DEX in DOG/DEX mice as compared with DOG/PBS mice (*p* > 0.99, Fig. 9a). In summary, our data show that DEX does not reverse the features of AR in our model.

## Discussion

In an original DOG-induced murine model of neutrophilic Th17-driven asthma, we investigated the response of airway inflammation and AR to high-dose of systemic DEX, a potent corticosteroid, in order to assess its relevance to mimic severe asthma. We found that DEX did not reduce AHR, AR-related smooth muscle hypertrophy and subepithelial fibrosis, as well as *Il17a* overexpression and IL-17A production. Neutrophilic airway inflammation, mucus hyperproduction and local humoral response, though improved, also persisted at significantly increased levels despite DEX. The only steroid fully-responsive feature was DOG-induced *Il22* overexpression which was completely abrogated.

The absence of full response to steroids of airway inflammation observed in our model aligns with clinical observations showing that neutrophilic inflammation and the T2^low^ endotype are associated with poor response to ICS [18, 23, 24]. Furthermore, the persistent inflammation despite steroids and the prominent induction of *Il17a* expression and production in lungs in our DOG-induced model are consistent with the well-documented association between IL-17 involvement and neutrophil inflammation in sputum, severe asthma, and poor response to steroids, observed in asthma patients [18, 23, 25]. Indeed, preclinical data show that IL-17 promotes steroid-resistant recruitment and activation of neutrophils in airways, by induction of chemokines and activating factors, like CXCL1, IL-8 and IL-6, by immune, endothelial, and epithelial cells [26–30]. Thus, in our study, the persistence of neutrophilic inflammation can be attributed to the steroid insensitivity of *Il17a* overexpression, leading to sustained neutrophil-chemoattractant chemokine *Cxcl1* expression. However, it should be noted that while CXCL1 levels in the lungs are not modified by DEX, the partial response of neutrophils to DEX suggests that other steroid-sensitive chemokines are likely involved in neutrophil recruitment in our model. Additionally, our results are in line with the findings of Stark et al. who reported corticosteroid-insensitive airway neutrophilia in another murine model of mixed Th2/Th17 asthma induced by nasal administration of DOG [29]. They confirmed the crucial role of IL-17 in DOG-induced airway neutrophilia, which was dramatically decreased by anti-IL-17 antibody treatment. The Mechanisms underlying the persistence of *Il17a* overexpression in steroid-treated mice might involve IL-6 and the transcription factor STAT3: both play a role in Th17 differentiation induction and have been showed to be insensitive to steroids themselves [30]. Besides, steroid-insensitivity of DOG-induced airway inflammation is consistent with several reported IL-17-induced mechanisms of steroid response impairment, including upregulation of the glucocorticoid receptor-β isoform [31], associated with steroid unresponsiveness, reduction of histone deacetylase 2 activity, which mediates transcriptional effects of steroids [32], or inhibition of *FKB5* and *HSD11B2* expressions, respectively involved in steroid affinity of glucocorticoid receptor and bioactivation of cortisol [33]. Moreover, in vivo and in vitro, a large number of IL-17A-induced genes and proteins are not inhibited (or even enhanced) by DEX, including neutrophilic inflammation-related cytokines [33].

One of the key strengths of our model is its ability to reproduce human AR. In this study, we found persistent AR features despite DEX treatment. This result is particularly significant, suggesting that our model successfully replicates the well-established AR observed in human T2^low^ severe asthma, associated with poor response to ICS [2, 11]. Furthermore, the persistence of mucus hyperproduction despite steroids aligns with findings from other in vitro and Th17 or mixed Th2/Th17-driven murine models [33–35], along with steroid-unresponsive subepithelial fibrosis [6, 35–37] and smooth muscle hypertrophy [35]. Notably, AHR is correlated with smooth muscle hypertrophy in T2^low^ asthma [38]. In this study, we also reported DEX-insensitive AHR in our DOG-induced model. This is consistent with studies in other animal models showing that Th17 response generates steroid-unresponsive AHR, and with the lack of inhibition of *Il13* expression in our work, a strong inducer of AHR [25, 34, 35, 39–41].

Interestingly, *Il22* expression was totally abolished by DEX in our study. This is in line with previous reports of IL-22 production steroid sensitivity, particularly in type 3 innate lymphoid cells [42, 43]. This result suggests that IL-22 does not mediate the steroid-insensitive features observed in our model. Given the role of IL-22 in epithelial repair [14], the lack of correlation between IL-22 and AR that we report in this study may seem inconsistent with a previous report from our group showing that downregulation of *Il22* expression in the DOG-induced murine model was associated with reduction in mucus hyperproduction and in airway smooth muscle hypertrophy [20]. One explanation is that in the previously-cited work, *Il22* downregulation was achieved by antagonization of the Aryl hydrocarbon receptor during the 3 consecutive weeks of allergen challenge, possibly before the establishment of AR. Improvement in smooth muscle hypertrophy could have also been mediated by other pathways downstream of this receptor. Therefore, the steroid sensitivity of IL-22 does not rule out its role in the establishment of AR.

One challenging issue in interpreting these results is the definition of what can be considered as steroid-resistant or not. Although there is a consensual definition of severe asthma in humans (lack of response to high-dose ICS plus another controller or the need for systemic steroids more than half a year) [8], no consensus has emerged on the definition of steroid resistance in asthma, whether for patients or animal models. It could correspond to a total lack of response to steroids, an insufficient or incomplete response, or to the need for high doses of steroids to obtain a response. Other pharmacological considerations are lacking for a complete definition in this setting, particularly the therapeutic molecule (DEX, prednisone, budesonide…), its administration route (inhaled versus systemic), the corresponding minimal dose, and the minimal treatment duration required to define steroid resistance. In this area, Gubernatorova et al. have referenced multiple murine models of asthma reported to be steroid-resistant or, at least, partially responsive [44]. Interestingly, of 14 studies that fully reported the details of steroid course, 12 (85%) used DEX and only 3 used more than 5 mg.kg^−1^ of cumulative dose, that is to say superior to the cumulative dose we used [34, 35, 39, 45–55]. Overall, it appears to us that an incomplete response to the cumulative dose of systemic DEX we used seems strict enough and relevant to properly conclude to steroid altered response in our model.

Lastly, while our model demonstrates several valuable features that mimic human pathology, it suffers from limitations inherent to murine models in transposability to human asthma. Beyond general biological differences between mice and humans, particularly in immune system, it is important to note that asthma does not spontaneously occur in mice, and our model replicates phenomena over a short timescale compared to the chronic progression seen in human asthma, especially for airway remodelling. The use of only female mice, chosen for consistency with the original model development and to reflect the predominance of women in allergic asthma, is a limitation that may influence generalizability. Additionally, C57BL/6 J mice, while commonly used for their well-characterized immune response, may have strain-specific responses that do not fully represent the heterogeneity of human populations. While our murine model serves as a useful tool for identifying potential therapeutic targets, translating these findings requires validation in patient-based research. Furthermore, the conclusions drawn from our protocol are specific to the configuration of the experimental design, including the timing of dexamethasone administration and the endpoints assessed. What is more, one limitation of our model and protocol is that it does not allow us to assess the reversal of the studied parameters by DEX during the final week of treatment. Instead, the findings primarily reflect the prevention of further worsening of these parameters induced by the last week of DOG challenge. To specifically evaluate the potential reversal of these parameters, a comparison of their status before and after the final week of the protocol, during which mice are treated with DEX, would be necessary.

In conclusion, our DOG-induced murine model of asthma is an innovant model, using a relevant allergen involved in human asthma, and exhibits no or only partial response to steroids of AR and Th17-associated neutrophilic inflammation. To our knowledge, this is the first model to demonstrate the lack of response to steroids of full features of AR (including mucus hyperproduction, subepithelial fibrosis and smooth muscle hypertrophy) in a T2^low^ inflammation context. Steroid insensitivity of AR and Th17 inflammation in this model underscore its significance for exploring AR in a T2^low^ severe asthma setting. By providing a robust platform to unravel the mechanisms underlying AR, a feature associated with poor outcomes in severe asthma (impaired quality of life, respiratory disability), this model offers promising opportunities to identify new therapeutic targets in the field of T2^low^ severe asthma-related AR and therefore alleviate the burden of the disease.

## Supplementary Information

Below is the link to the electronic supplementary material.

Supplementary file 1 (EPS 82 KB)

Supplementary file 2 (DOCX 53 KB)

## Data Availability

No datasets were generated or analysed during the current study.

## Abbreviations

AHR: Airway hyperresponsiveness
AR: Airway remodelling
BAL: Bronchoalveolar lavage
DEX: Dexamethasone
DOG: Dog allergen
H&E: Haematoxylin an Eosin staining
ICS: Inhaled corticosteroids
IL: Interleukine
Ig: Immunoglobulin
IR: Index of reactivity
MPO: Myeloperoxydase
PAS: Periodic acid-schiff
PBS: Phosphate buffered saline
