# Supplementary material for: Dog allergen-induced asthma in mice: a relevant model of T2low severe asthma with airway remodelling
Source: Inflamm Res. 2025 Mar 14;74(1):52. doi: 10.1007/s00011-025-02004-9 (PMC11906515; doi:10.1007/s00011-025-02004-9)
Supplement: Supplementary file 2 — (DOCX 53 KB) [file 11_2025_2004_MOESM2_ESM.docx]

**Supplementary dataS1**

**Dog allergen-induced asthma in mice: a relevant model of T2^low^ severe asthma with airway remodelling**

**Journal:** Inflammation Research

**Authors**

Victor Margelidon-Cozzolino^1,2*^, Joanne Balsamelli^1^, Saliha Ait-Yahia^1^, Marie-Hélène Gevaert^3^, Silvia Demoulin-Alexikova^1^, Muriel Pichavant^1^, Anne Tsicopoulos^1^, Cécile Chenivesse^1,4^, Stéphanie Lejeune^1,5^, Patricia de Nadai^1^

**Affiliations**

^1^ Univ. Lille, CNRS, Inserm, CHU Lille, Institut Pasteur de Lille, U1019 - UMR9017 - CIIL-Center for Infection and Immunity of Lille, F-59000, Lille, France

^2^ Groupement des Hôpitaux de l’Institut Catholique de Lille (GHICL), Lille, France.

^3^ CNRS, Inserm, CHU Lille, Univ. Lille, Institut Pasteur de Lille, US 41-UAR 2014-PLBS, Lille, France

^4^ CRISALIS (Clinical Research Initiative In Severe Asthma: a Lever for Innovation & Science), F-CRIN network, INSERM US015, Toulouse, France

^5^ Univ. Lille, Department of Pediatric Pulmonology and Allergy, Hôpital Jeanne de Flandre, CHU Lille, F-59000, Lille, France.

***Correspondence**Victor Margelidon-Cozzolino

Service de Pneumologie, Hôpital Saint-Philibert, rue du Grand But, 59160 Lomme

[margelidon.victor@ghicl.net](mailto:margelidon.victor@ghicl.net)

Tel: +33 3 20 87 71 83

Fax: +33 (0)3 20 87 73 45

**Supplemental Material**

**Material and reagents**

Methacholine was purchased from Sigma-Aldrich (St Louis, MO, USA), medetomidine (DOMITOR) from Vetoquinol (Magny-Vernois, France), ketamine (IMALGENE) and isoflurane (VETFLURANE) from Virbac (Carros, France), pentoparbital (EUTHASOL) from Centravet (Dinan, France).

May-Grünwald Giemsa staining kit, Antigenfix and paraffin (Histowax) were purchased from Microm Microtech (Brignais, France). Haematoxylin and Eosin, Periodic Acid-Schiff and Masson’s Trichrome staining kits were purchased from Biognost (Zagreb, Croatia). Cytocentrifugation was performed with Shandon cytospin 4 (ThermoFisher Scientific, Waltham, USA). The mouse-on-mouse kit-AP detection system with permanent Red was purchased from Neobiotech (Nanterre, France).

For ELISAs, biotinylated goat anti-IgE antibody was purchased from Gentaur (Paris, France), anti-IgG_1_ antibody from ThermoFisher Scientific (Illkirch, France), TMB substrate solution from Sigma-Aldrich (Saint-Quentin Fallavier, France). The mouse MPO an total IgA commercial ELISA kits were purchased from Invitrogen (Waltham, USA). IL-22 was quantified in bronchoalveolar lavage using a commercial kit (R & D Systems, Minneapolis, USA). IL-22 was quantified in bronchoalveolar lavage (BAL) fluid using a commercial kit (R&D Systems, Minneapolis, USA). IL-4, IL-13, IL-17A, and CXCL1 were simultaneously quantified using a multiplex ELISA kit (U-PLEX, MSD, Maryland, USA). Total lung proteins were quantified with the Pierce BCA Protein Assay Kit (Life Technologies, Carlsbad, USA). Proteins were extracted from total lung tissue by mechanically dissociating a pulmonary lobe in 1 mL of lysis buffer (Tissue-Protein Extraction Reagent, Life Technologies, Carlsbad, USA) supplemented with a protease inhibitor cocktail (Sigma Aldrich, Saint-Quentin-Fallavier, France) at 4°C, followed by centrifugation to collect the supernatant.

**Semi-quantitative scoring of mucus production and subepithelial fibrosis**

Mucus production

Before embedding, the lungs were divided into three parts, representing the superior, middle, and inferior regions, each yielding a distinct lung section on a slide corresponding to one mouse. After PAS staining, an intensity score for mucus production was assigned to each bronchus of all lung sections from each mouse, according to the semi-quantitative scale given in Supplementary Table S1. For each mouse, an arithmetic mean value of all bronchial scores was calculated and used for statistical analysis.

Subepithelial fibrosis

Following the same cutting and embedding process as previously described, each slide containing three lung sections per mouse was stained with Masson’s Trichrome. Similar to the assessment of mucus production, an intensity score for subepithelial fibrosis was assigned to each bronchus in all lung sections, according to the semi-quantitative scale detailed in Supplementary Table S2. The arithmetic mean of all bronchial scores for each mouse was calculated and used for statistical analysis.

**SUPPLEMENTARY TABLES**

**Supp. Tab. S1: Semi-quantitative scale of mucus production scoring**

| **Bronchus Score** | **Corresponding description** |
| --- | --- |
| 0 | \| No mucus \| \| --- \| |
| 1 | \| Less than 25% of epithelial cells are mucus-positive \| \| --- \| |
| 2 | \| Between 25% and 50% of epithelial cells are mucus-positive \| \| --- \| |
| 3 | \| Between 50% and 75% of epithelial cells are mucus-positive \| \| --- \| |
| 4 | \| Between 75% and 100% of epithelial cells are mucus positive \| \| --- \| |

**Supp. Tab. S2: Semi-quantitative scale of subepithelial fibrosis scoring**

| **Bronchus Score** | **Corresponding description** |
| --- | --- |
| 1 | \| No observable collagen deposition beneath the epithelial layer \| \| --- \| |
| 2 | \| Discontinuous and thin collagen deposition beneath the epithelial layer \| \| --- \| |
| 3 | \| Thin, continuous and consistent collagen deposition beneath the epithelial layer around the entire bronchial circumference \| \| --- \| |
| 4 | \| Dense and thick collagen deposition beneath the epithelial layer around the entire bronchial circumference \| \| --- \| |

**Supp. Tab. S3: Prime time assay references used for the quantitative PCR**

| **Target** | **Catalogs** |
| --- | --- |
| *Col24a1* | Mm.PT.58.29594619 |
| *Cxcl1* | Mm.PT.58.42076891 |
| *Ifnγ* | Mm.PT.58.41769240 |
| *Il4* | Mm.PT.58.7882098 |
| *Il13* | Mm.PT.58.31366752 |
| *Il17a* | Mm.PT.58.6531092 |
| *Il17f* | Mm.PT.58.9739903 |
| *Il22* | Designed primers & probe:   - Primer 1 : 5’-GACTCCTCGGAACAGTTTCTC-3’ - Primer 2 : 5’-AGCTTGAGGTGTCCAACTTC - 3’ - Probe :   5’- /56-FAM/ACATCGTCA/ZEN/ACCGCACCTTTATGC/3IABkFQ/- 3’ |
| *Muc5ac* | Mm.PT.58.42279692 |
| *Muc5b* | Mm.PT.58.30457752 |
| *Rplp0* | Mm.PT.58.43894205 |

**Supplemental Results and Figures**

**Quantification of IL-22 in BAL fluid**

To further validate the complete suppression of *Il22* gene expression we observed in response to dexamethasone, we quantified IL-22 in BAL fluid (Supp. Fig. S1). The rise of IL-22 level induced by DOG allergen in the DOG/PBS group was abolished by dexamethasone, as observed in the DOG/DEX group.


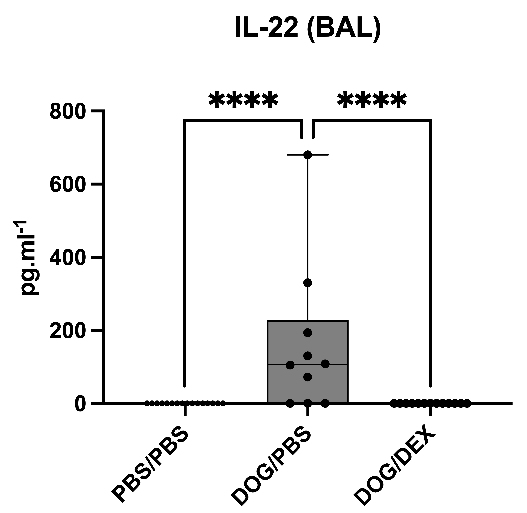


**Supp. Fig. S1** Quantification of IL-22 in BAL fluid. Results are expressed in pg.ml^-1^, as median, interquartile range, minimum and maximum; n= 10-16 per group; Kruskall-Wallis tests, ****p<0.0001. Ruled-out outliers: 3 in DOG/PBS; 5 in DOG/PBS. Experiments were performed 3 times
